# Strategies for adaptation to high light in plants

**DOI:** 10.1007/s42994-024-00164-6

**Published:** 2024-05-13

**Authors:** Man Zhang, Yu Ming, Hong-Bin Wang, Hong-Lei Jin

**Affiliations:** 1 State Key Laboratory of Traditional Chinese Medicine/School of Pharmaceutical Sciences, Guangzhou University of Chinese Medicine, Guangzhou, 510006 China; 2 Institute of Medical Plant Physiology and Ecology, Guangzhou University of Chinese Medicine, Guangzhou, 510006 China; 3 Key Laboratory of Chinese Medicinal Resource From Lingnan (Guangzhou University of Chinese Medicine), Ministry of Education, Guangzhou, 510006 China; 4 Guangzhou Key Laboratory of Chinese Medicine Research on Prevention and Treatment of Osteoporosis, The Third Affiliated Hospital of Guangzhou University of Chinese Medicine, Guangzhou, 510375 China

**Keywords:** Defense, Photoprotection, Photosystem, Repair of PSII

## Abstract

Plants absorb light energy for photosynthesis via photosystem complexes in their chloroplasts. However, excess light can damage the photosystems and decrease photosynthetic output, thereby inhibiting plant growth and development. Plants have developed a series of light acclimation strategies that allow them to withstand high light. In the first line of defense against excess light, leaves and chloroplasts move away from the light and the plant accumulates compounds that filter and reflect the light. In the second line of defense, known as photoprotection, plants dissipate excess light energy through non-photochemical quenching, cyclic electron transport, photorespiration, and scavenging of excess reactive oxygen species. In the third line of defense, which occurs after photodamage, plants initiate a cycle of photosystem (mainly photosystem II) repair. In addition to being the site of photosynthesis, chloroplasts sense stress, especially light stress, and transduce the stress signal to the nucleus, where it modulates the expression of genes involved in the stress response. In this review, we discuss current progress in our understanding of the strategies and mechanisms employed by plants to withstand high light at the whole-plant, cellular, physiological, and molecular levels across the three lines of defense.

## Introduction

During their growth and development, plants are affected by many environmental factors, including light, which is an important environmental cue (Yadav et al. 2020). Plants absorb light energy via their photosystems in chloroplasts to initiate photosynthesis. However, excess light energy can damage the photosynthetic apparatus and diminish photosynthetic efficiency. Absorption of excess light leads to increased production of excited, highly active photosynthetic intermediates, which puts plants at risk of severe photodamage (Pinnola and Bassi 2018; Shi et al. 2022). Therefore, it is particularly important to study how plants withstand high light.

**Fig. 1:**
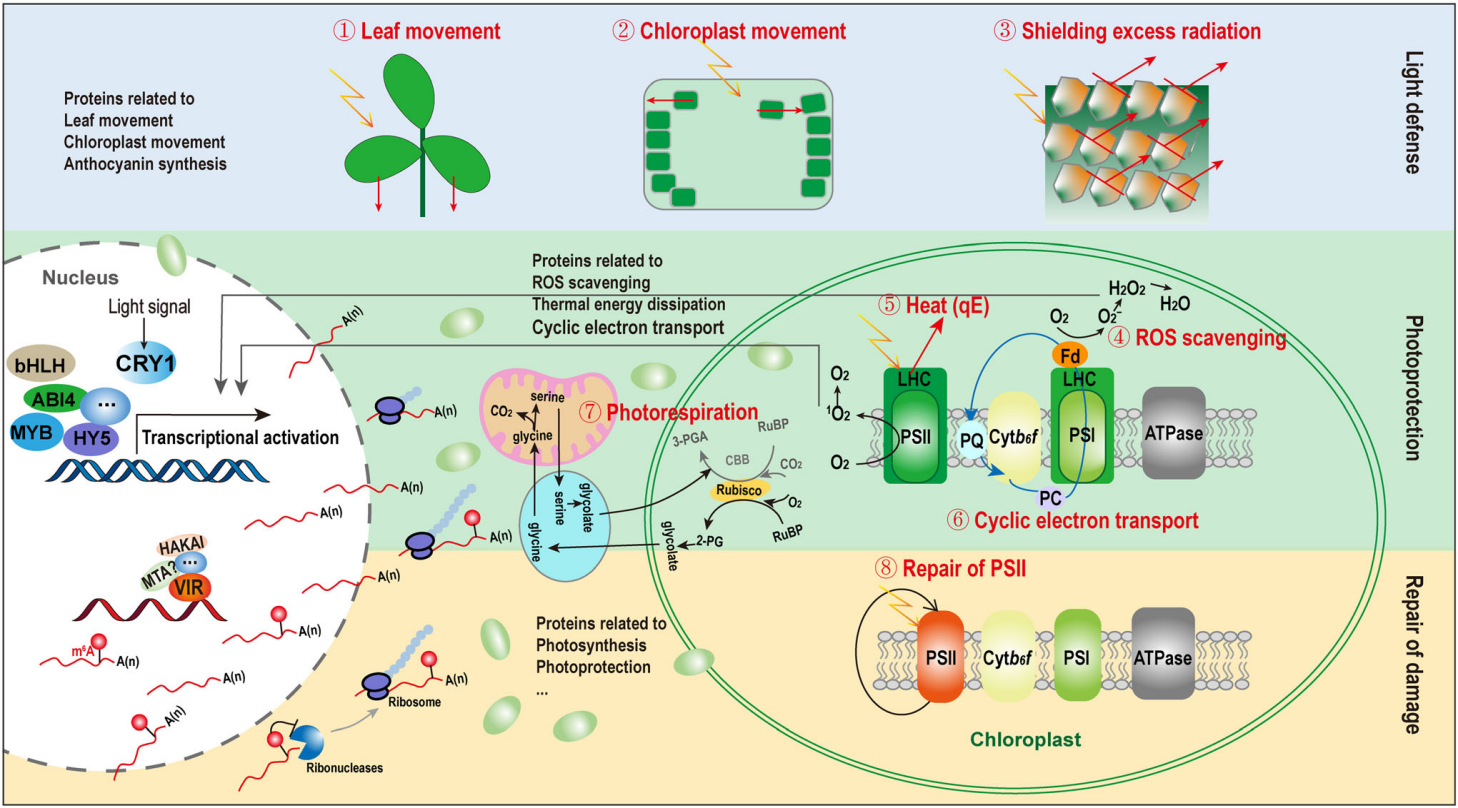
Photoprotection mechanisms in plants. Plants have developed three main lines of defense to respond to excess light. The first line of defense, avoidance, includes avoiding exposure to light through leaf and chloroplast movement and shielding excess radiation via the production of secondary metabolites. The second line of defense, photoprotection, involves ROS scavenging, dissipation of excessive energy as heat, CET, and photorespiration. The last line of defense, PSII repair, repairs the damage caused by high light. *CBB* Calvin-Benson-Bassham cycle, *3-PGA* 3-phosphoglyceric acid, *2-PG* 2-phosphoglycolate

When plants are subjected to high light, the energy distribution between photosystem I (PSI) and PSII becomes unbalanced, leading to a sharp drop in photosynthetic efficiency. As a result, the plant may eventually wither and die. In response to this type of stress, plants employ a variety of self-protection mechanisms that form three lines of defense. When a plant is exposed to high light, the first line of defense is initiated, in which leaves and chloroplasts move away from the light and the plant accumulates compounds that filter and reflect the light to limit the absorption of excessive light energy. The second line of defense protects the photosystems by dissipating excess light energy and thereby limiting the oxidative damage caused by reactive oxygen species (ROS). If the coordinated effects of the first two lines of defense do not protect the plant from a certain degree of damage, the plant must activate the third line of defense: repair of PSII. Although a relatively low level of repair occurs even under normal light conditions, the speed of the repair cycle greatly increases after photodamage (Bassi and Dall'Osto 2021; Moejes et al. 2017; Pinnola and Bassi 2018; Shi et al. 2022; Takahashi and Badger 2011). In this review, we discuss the current knowledge of these three main lines of photoprotective defense used by plants (Fig. 1).

## First line of defense: limiting the absorption of excess light

### Leaf and chloroplast movement

Many plants respond to external light intensity by altering leaf angle. Under low-light conditions, leaves move toward the light source, forming a perpendicular angle to the incident light, which maximizes light energy absorption. Under high-light conditions, plants adjust the position and angle of their leaves to be parallel to the direction of light, decreasing their absorption of light energy and lowering leaf temperature (Murchie and Niyogi 2011; Takahashi and Badger 2011). For example, exposure to higher light intensity decreases the abaxial leaf petiole angle and increases the light absorption area and photosynthetic activity of soybean (*Glycine max*) plants (Feng et al. 2019). Paraheliotropism, the orientation of leaves parallel to light rays, appears to be an important mechanism by which the common bean (*Phaseolus vulgaris*) avoids photoinhibition (Pastenes et al. 2005). At midday, vertically oriented leaves in rice (*Oryza sativa*) undergo less photoinhibition than horizontal leaves (Murchie et al. 1999). Other environmental conditions also affect leaf movement, such as temperature, humidity, and nutrient availability.

At the cellular level, chloroplasts optimize the capture of light used for photosynthesis by changing their positions within the cell. Under low-light conditions, chloroplasts absorb more light energy by aggregating at the cell surface. In high-light environments, chloroplasts avoid absorbing excess light energy by moving to the anticlinal cell wall, thus increasing the amount of light transmitted through the leaf. Photosynthesis occurs at the individual chloroplast level; therefore, the subtle subcellular movement of these organelles can accurately respond to small changes in light intensity (Murchie and Niyogi 2011). The photoreceptors phototropin1 (phot1) and phot2 function in chloroplast movement, playing important roles in phototaxis in *Arabidopsis* (*Arabidopsis thaliana*). The high-light-avoidance response is mainly regulated by phot2 (Ishishita et al. 2020).

Actin filaments along the chloroplast periphery on the plasma membrane side provide the motive force needed for chloroplast movement (Kadota et al. 2009). Several actin filament regulators participate in actin-regulated chloroplast movement. Chloroplast unusual positioning1 (CHUP1) anchors chloroplasts to the plasma membrane in *Arabidopsis*; the chloroplasts of *chup1* mutants do not move in response to light and instead aggregate at the bottom of the cell irrespective of light conditions (Suetsugu and Wada 2008; Oikawa et al. 2003). Kinesin-like protein for actin-based chloroplast movement (KAC) family proteins play vital roles in the movement of chloroplasts and their attachment to the plasma membrane. Chloroplast photorelocation is disrupted in the *Arabidopsis kac1 kac2* double mutant, and the chloroplasts are detached from the plasma membrane (Suetsugu et al. 2010). Other factors are also critical for chloroplast movement, such as THRUMIN1 and Plastid movement impaired1 (Dwyer and Hangarter 2022).

### Shielding plant cells from excess light radiation

During their long-term adaptation to light, plants have evolved a photodamage defense mechanism: When exposed to strong light or ultraviolet (UV) radiation, plants quickly accumulate phenolic compounds, including phenolic acids, flavonols, flavones, and anthocyanins, inside leaf cells. These compounds act as sunscreens that filter and reflect excessive radiation (Araguirang and Richter 2022). Phenolic compounds most commonly occur in vacuoles of epidermal or outermost mesophyll cells. Some flavonols and flavones, such as quercetin, kaempferol, and apigenin, have an absorption peak in the UV region (Ferreyra et al. 2021). Anthocyanins, one of the most widely distributed flavonoids in plants, not only give plants their bright colors but also play important roles in plant adaptation to abiotic stress, especially in high-light adaptation. Anthocyanins absorb visible light as well as some UV light in the solar spectrum (Takahashi and Badger 2011). Following their acetylation with phenylpropanoid acids, the acylated derivatives of anthocyanins have enhanced UV light absorbance (Ferreyra et al. 2021). Carotenoids and alkaloids (betalains) in higher plants, as well as mycosporine-like amino acids in lower plants, are also photoprotective pigments that attenuate radiation in the visible and UV spectra (Solovchenko and Merzlyak 2008).

High light and UV radiation induce the biosynthesis of phenolic compounds in plants (Araguirang and Richter 2022; Lingwan et al. 2023). PSII is one of the main targets of high light– and UV radiation–induced damage. The light-shielding effect of phenolic compounds helps protect PSII from photodamage (Agati et al. 2013; Ferreyra et al. 2021). Indeed, high-altitude plants activate flavonoid biosynthesis pathways and accumulate more flavonoids to adapt to high light and increased UV radiation compared with low-altitude plants (Sharma et al. 2019). Red poinsettia leaves (containing high levels of anthocyanins) had significantly higher PSII quantum efficiency with less photoinhibition than green leaves under excessive light irradiation (Moustaka et al. 2018). Upregulating the biosynthesis of flavonoids enhanced the tolerance to UV light and high light compared to wild-type plants (Peng et al. 2017; Righini et al. 2019; Zhang et al. 2022a). Furthermore, double *pal1 pal2* mutants impaired in the early steps of phenolic compound biosynthesis show enhanced sensitivity to UV light, the reduced capacity of PSII photochemistry, and compromised photoprotection under UV light treatment compared to wild-type plants (Huang et al. 2010). In addition to being activated under high light and UV light, phenolic compounds, as an important class of plant secondary metabolites, also help plants acclimatize to a wide range of unfavorable environments such as drought, salinity, and high/low-temperature stresses, and they play crucial roles throughout the plant life cycle (Sharma et al. 2019). Therefore, enhancing the biosynthesis of phenolic compounds can be strategically utilized to enhance plant stress tolerance (Lingwan et al. 2023).

## Second line of defense: photoprotection

### Thermal energy dissipation

Under normal illumination, the light-harvesting complex captures light energy and transitions chlorophyll molecules from the ground state to the singlet excited state. The resulting excitation energy is transferred to the photosystem reaction centers to drive photochemical reactions, and the singlet chlorophyll molecule then returns to the ground state. When plants are exposed to light levels that exceed energy demands, chlorophyll molecules are overexcited and accumulate excess excitation energy in photosynthetic membranes, which can be harmful to PSII. Return of excited chlorophyll molecules to the ground state occurs mainly via three mechanisms: (1) photochemical quenching, which drives photochemical reactions (e.g., photosynthesis); (2) fluorescence quenching, which dissipates excess excitation as chlorophyll fluorescence; and (3) non-photochemical energy quenching (NPQ), which harmlessly dissipates excess excitation energy in the PSII antenna complexes as heat (Baker 2008). NPQ is the fastest response of photosynthetic membranes to excess light (Demmig-Adams et al. 2014; Ruban 2016).

NPQ involves several components that can be distinguished by their recovery kinetics: the state transition (qT), photoinhibition-dependent quenching (qI), zeaxanthin-dependent quenching (qZ), sustained quenching (qH), and ΔpH or energy-dependent non-photochemical quenching (qE). The state transition (qT) displaces the light-harvesting complex II (LHCII) antenna between PSII and PSI to redistribute the excitation energy between the two photosystems (Bellafiore et al. 2005). This step is important in algae but is rather negligible in most plants during exposure to excess light (Allorent et al. 2013; Müller et al. 2001). Photoinhibition-dependent quenching (qI) is caused by photoinhibition and shows very slow relaxation kinetics in the range of hours (Müller et al. 2001).

Zeaxanthin-dependent quenching (qZ) relies on zeaxanthin and is activated within tens of minutes; this process likely forms in the antenna of PSII at those xanthophyll binding sites, which are slowly converted to zeaxanthin (Nilkens et al. 2010). Sustained quenching (qH) is a very slow component of NPQ (Brooks et al. 2013) that is independent of known components required for other types of NPQ, such as ΔpH, PsbS, zeaxanthin, or the kinase state transition7 (STN7). qH occurs in the antenna, specifically in the peripheral antenna of PSII (Malnoë et al. 2018). The photoprotective mechanism of qH requires lipocalin in the plastid (LCNP) and is prevented by the suppressor of quenching1 (SOQ1). Relaxation of QH1 (ROQH1) functions as a qH relaxation factor. LCNP and ROQH1 are proposed to play dosage-dependent, antagonistic functions in protecting the photosynthetic apparatus and maintaining light-harvesting efficiency in plants (Amstutz et al. 2020). Hypersensitive to high light1 (HHL1), a damage repair factor for PSII (Jin et al. 2014), was recently shown to interact with SOQ1 and synergistically regulate qH (Duan et al. 2023).

ΔpH or energy-dependent non-photochemical quenching (qE) is the main form of heat dissipation that plays a photoprotective role in plants and algae (Buck et al. 2019; Goss and Lepetit 2015; Niyogi and Truong 2013). Excess light leads to increased ΔpH in the thylakoids produced by photosynthetic electron transfer. A decrease in pH in the thylakoids serves as an immediate signal of excessive light, triggering feedback regulation of light capture by qE. qE is rapidly reversible and can be induced or eliminated within a few seconds, thereby quickly responding to fluctuations in light intensity in the natural environment (Goss and Lepetit 2015). Low pH in the thylakoid lumen activates violaxanthin de-epoxidase (VDE), which converts violaxanthin to antheraxanthin and zeaxanthin; these compounds are required for qE formation. In addition to requiring zeaxanthin, qE requires the presence of the protein PsbS. PsbS serves as a pH sensor that detects thylakoid lumen acidification and transduces signals to the antenna, playing an important role in regulating the rapid induction and relaxation of qE (Correa-Galvis et al. 2016; Krishnan-Schmieden et al. 2021; Li et al. 2004). The response of PsbS to pH has been revealed to be a functional conformational switch (Chiariello et al. 2023; Krishnan-Schmieden et al. 2021). The transcription factors OsbZIP72 and OsMYBS2 play reversible roles in synergistically regulating *OsPsbS1* transcription in rice (Fu et al. 2021). PsbS-dependent NPQ primarily occurs in LHCII, as an *Arabidopsis* mutant lacking LHCII exhibits approximately 60% lower NPQ than the wild type (Nicol et al. 2019). Accelerating the xanthophyll cycle and increasing PsbS levels lead to faster induction and relaxation of NPQ, which increases the photosynthetic efficiency of plants under fluctuating light conditions and ultimately improves crop yield (De Souza et al. 2022; Kromdijk et al. 2016).

### Cyclic electron transport

Photosynthetic electron transport comprises two major pathways: linear electron transport (LET) and cyclic electron transport (CET) (Munekage et al. 2004). LET mediates electron transport through PSII, cytochrome *b*_*6*_*f* (Cyt *b*_*6*_*f*), and PSI to NADP^+^ to produce NADPH while forming the proton gradient across the thylakoid membrane needed to drive ATP synthase to produce ATP. CET around PSI does not involve PSII. The electrons transferred to PSI are ultimately returned to Cyt *b*_*6*_*f* without generating NADPH; only the ΔpH across the thylakoid membrane is generated for ATP biosynthesis (Yamori and Shikanai 2016).

Angiosperms typically employ two distinct CET pathways: a major pathway that depends on proton gradient regulation5 (PGR5) and PGR5-like photosynthetic phenotype1 (PGRL1) and a minor pathway, the chloroplast NADH dehydrogenase-like (NDH) complex-dependent electron transport pathway. Indeed, the *pgr5* and *pgrl1* mutants in *Arabidopsis* exhibit hypersensitivity to high light (DalCorso et al. 2008; Munekage et al. 2002). CET has important physiological significance, as it adjusts the ATP/NADPH ratio in chloroplasts to meet the demands of the Calvin-Benson-Bassham cycle. CET also plays a vital role in photoprotection: CET-induced acidification of the chloroplast lumen regulates photosynthetic electron transport, inducing the qE component of NPQ to dissipate excess absorbed light energy, thereby protecting PSII and PSI (Murchie and Niyogi 2011; Niu et al. 2023; Yamori and Shikanai 2016) and helping photosynthetic organisms respond quickly to environmental changes.

PGR5 plays crucial roles in PSI photoprotection under fluctuating light conditions (Suorsa et al. 2012; Yamamoto and Shikanai 2019). In the *pgr5* mutant, photodamage occurs in PSI before it occurs in PSII, and the mutant cannot survive under naturally or artificially fluctuating light conditions (Suorsa et al. 2012). PGR5 can operate in CET on its own, but its activity must be modulated by PGRL1. PGRL2 can interact with PGR5 and PGRL1 and negatively regulates the stability of PGR5 (Rühle et al. 2021).

NDH is a very large thylakoid membrane protein complex with multiple subunits. The NDH complex contains at least 29 protein subunits and associates with PSI to form an NDH–PSI supercomplex, which helps stabilize the NDH complex and facilitates CET (Shen et al. 2022; Su et al. 2022). NDH-dependent CET is involved in plant responses to various types of environmental stresses, including high light, low humidity, drought, and abnormal temperatures (Yamori and Shikanai 2016). The NDH complex alleviates oxidative stress in chloroplasts under excess-light conditions. Tobacco (*Nicotiana tabacum*) *ndhB* mutants lack qE and exhibit hypersensitivity to high light (Endo et al. 1999; Horváth et al. 2000). NDH-dependent CET around PSI also plays important roles in efficient electron transport at low light intensities and photoprotection of PSI under fluctuating light conditions in rice (Yamori and Shikanai 2016). NDH-defective mutants show a concomitant decrease in CO_2_ assimilation rate and plant biomass under low light intensities, as well as PSI photoinhibition and diminished plant growth under fluctuating light conditions (Yamori et al. 2016).

### Photorespiration

Photorespiration is a complex light-dependent set of reactions during which plants take up O_2_, accompanied by the release of CO_2_. Ribulose-1,5-bisphosphate carboxylase/oxygenase (Rubisco) has dual activities: carboxylation and oxygenation. At relatively high CO_2_ concentrations, Rubisco catalyzes the carboxylation of ribulose-1,5-bisphosphate (RuBP) to produce two molecules of 3-phosphoglyceric acid (3-PGA) that are integrated into the Calvin-Benson-Bassham cycle. When CO_2_ is limited, Rubisco catalyzes the oxygenation of RuBP to produce one molecule of 3-PGA and one molecule of 2-phosphoglycolate (2-PG). The phosphate group is removed from 2-PG to generate glycolate, which is then metabolized to glycine in the peroxisome and to serine in the mitochondrion along with CO_2_ release. Serine is subsequently converted back to 3-PGA in the photorespiratory cycle (Bauwe et al. 2010; Hou et al. 2019; Peterhansel et al. 2010).

During photorespiration, ATP and reducing (NAD(P)H) equivalents are consumed, and ammonia (NH_3_) and CO_2_ are released. This metabolic pathway is often viewed as wasteful (Shi and Bloom 2021; Wingler et al. 2000). However, suppressing or disrupting photorespiration does not improve plant net photosynthetic efficiency, and photorespiration mutants exhibit severe growth inhibition or a conditional lethal phenotype (Pick et al. 2013; Timm and Bauwe 2013; Voll et al. 2006). Photorespiration acts as a safety valve when the energy pressure on the photosynthetic apparatus increases, preventing overreduction of the photosynthetic electron transport chain and photoinhibition (Peterhansel et al. 2010; Wingler et al. 2000). Impairment of the photorespiratory pathway accelerates the photoinhibition of PSII by suppressing repair but not accelerating damage in *Arabidopsis* (Takahashi et al. 2007). Photorespiration limits CO_2_ fixation and reduces photosynthetic efficiency; the estimated loss due to photorespiration can reach 20–50% in C3 plants (South et al. 2019). Therefore, photorespiration has long been a core target for enhancing crop productivity through biotechnology (Fernie and Bauwe 2020; Walker et al. 2016). Engineering a photorespiration bypass with lower energy costs to recycle the toxic intermediate metabolite glycolate, rather than inhibiting photorespiration, has been successfully applied in plants, significantly increasing their photosynthetic efficiency and productivity (Eisenhut et al. 2019; Shen et al. 2019; South et al. 2019).

### ROS scavenging

In addition to being harmful by-products of aerobic metabolism that are continuously produced in plants, ROS are regulatory molecules that play important roles in signal transduction under high-light conditions (Exposito-Rodriguez et al. 2017; Foyer and Hanke 2022; Foyer and Noctor 2003). In thylakoids, the PSI and PSII reaction centers are the major sites of ROS generation. The ROS produced by chloroplasts include superoxide anion radical (O_2_^−^), hydrogen peroxide (H_2_O_2_), hydroxyl radicals (·OH), and singlet oxygen (^1^O_2_) (Gururani et al. 2015). Under excessive light irradiation, overexcitation of PSII promotes the formation of chlorophyll in the triplet excited state, which excites oxygen to generate ^1^O_2_. In PSI, oxygen receives electrons to generate O_2_^−^ and further generates H_2_O_2_ and ·OH (Asada 2006; Li et al. 2009). Under normal light and physiological metabolic conditions, the production and elimination of ROS are in a steady-state equilibrium, while under high-light conditions, the production of ROS at PSII and PSI in chloroplasts increases substantially. ROS are highly active substances that can cause photoinhibition by directly damaging chloroplast membrane systems. ROS can also accelerate photoinhibition by disrupting the repair of photodamaged PSII (Takahashi and Badger 2011). ROS accumulation in chloroplasts causes irreversible damage to the PSII core protein D1 and inhibits de novo biosynthesis of new D1 (Nishiyama et al. 2004, 2001). The inhibition of de novo D1 protein biosynthesis occurs at the translational level, revealing that ROS affects the activity of translation elongation factors (Kojima et al. 2007).

To avoid excessive ROS accumulation, a complex ROS-scavenging antioxidant network degrades ROS in chloroplasts, thereby minimizing photooxidative damage. ROS scavenging occurs via enzymatic and non-enzymatic antioxidant defense pathways. Antioxidant enzymes include superoxide dismutase (SOD), catalase (CAT), peroxidases, ascorbate peroxidase (APX), glutathione peroxidase, glutathione reductase (GR), monodehydroascorbate reductase (MDHAR), dehydroascorbate reductase (DHAR), and guaiacol peroxidase (GPX). SODs decompose O_2_^−^ to H_2_O_2_, while CATs catalyze the dismutation of H_2_O_2_ to H_2_O and O_2_, which is predominantly produced during photorespiration.

Non-enzymatic antioxidant metabolites, such as ascorbate, glutathione, tocopherols, and carotenoids, are also important for ROS scavenging (Bassi and Dall’Osto 2021; Takahashi and Badger 2011). The ascorbate–glutathione cycle helps maintain the balance of cellular H_2_O_2_ levels. APX reduces H_2_O_2_ to H_2_O and monodehydroascorbate (MDHA) using ascorbate as a reducing agent. MDHA can be reversibly reduced by MDHAR back to ascorbate. Dehydroascorbate (DHA) is reduced to ascorbate by DHAR using glutathione (GSH) as an electron donor. Glutathione disulfide (GSSG) is then reduced to GSH by GR using NADPH as a reductant (Chapman et al. 2019; Das and Roychoudhury 2014; Dvořák et al. 2021).

## Third line of defense: repair of damaged psii

When the synergistic effects of the above photoprotection pathways are not sufficient to avoid photodamage to PSII, plants activate the PSII repair system to quickly restore damaged PSII. Rapid repair of PSII also protects PSI from irreversible damage (Tikkanen et al. 2014). There are two major aspects of PSII repair: disassembly and reassembly of PSII complexes (Mulo et al. 2008).

PSII is a multi-subunit pigment–protein complex composed of dozens of protein subunits and hundreds of cofactors. The PSII core complex is composed of reaction center proteins and peripheral proteins arranged loosely around PSII (including oxygen-releasing complexes located in the thylakoid lumen). PSII reaction center proteins include the chloroplast genome–encoded D1, D2, CP43, and CP47, which play crucial roles in maintaining PSII function. PSII core complexes typically exist as dimers and combine with LHCII antennae to form the PSII supercomplex, a large supramolecular complex mainly localized in strictly stacked grana thylakoid regions. In these regions, only a very small stromal gap remains, where bulky proteases and ribosomes cannot enter (Kirchhoff 2014). Therefore, the photodamaged PSII needs to be exposed in the stroma lamellae. To allow photodamaged photosynthetic proteins in the PSII supercomplex to have access to the repair machinery, the supercomplex must first disassemble and release PSII monomers into the stroma lamellae (Järvi et al. 2015; Johnson and Pakrasi 2022; Nixon et al. 2010; Su et al. 2023; Theis and Schroda 2016).

### Disassembly of PSII complexes

After high-light-induced photodamage, the kinase STN7 phosphorylates LHCII, mediating its dissociation from PSII and binding to PSI, thereby balancing the energy distribution between PSII and PSI (Pesaresi et al. 2009). In contrast to STN7, Phosphatase1 (PPH1, also named Thylakoid-associated phosphatase38 [TAP38]) is responsible for the dephosphorylation of LHCII (Pribil et al. 2010; Shapiguzov et al. 2010). Phosphorylation of core PSII proteins is mainly achieved through the kinase STN8, and the phosphorylated PSII monomers migrate from grana stacks to stroma membranes (repair sites). The PSII core monomer is then dephosphorylated by PSII core phosphatase (PBCP) (Samol et al. 2012) and partially disassembles, releasing CP43 and the oxygen-evolving complex (OEC) to generate a CP43-free PSII monomer (Theis and Schroda 2016).

Subsequently, the PSII core proteins that were damaged by photooxidative stress are rapidly degraded. Two types of chloroplast proteases synergistically degrade the photodamaged D1 protein: members of the Deg family of serine-type ATP-independent proteases and the FtsH family of ATP-dependent zinc metalloproteases (Su et al. 2023; Theis and Schroda 2016; Yoshioka-Nishimura 2016). Thylakoid Lumen Protein of 18.3 kDa (TLP18.3), which interacts with Deg1, functions as an auxiliary protein that assists in D1 degradation and the dimerization of PSII complexes (Sirpiö et al. 2007; Zienkiewicz et al. 2012).

In addition, high light induces the partial unstacking and bending of stacked thylakoid membranes, accompanied by thylakoid swelling, which promotes contact of Deg proteases with damaged PSII core proteins and helps FtsH access the D1 protein in the grana region (Kirchhoff 2014; Yoshioka-Nishimura 2016). The dynamic structural changes in the thylakoid membrane are crucial for initiating efficient PSII repair. Plants that lack Curvature thylakoid1 (CURT1) show less adjustment of grana diameter and impaired membrane curvature at the grana margins compared with wild-type plants, resulting in a compromised PSII repair cycle (Pribil et al. 2018). Atomic force microscopy and scanning electron microscopy studies have also revealed that the thylakoid membrane system can undergo massive structural reorganization (Chuartzman et al. 2008; Kirchhoff 2014).

### Reassembly of PSII complexes

After the damaged D1 protein is degraded, a new functional copy is de novo synthesized and inserted into PSII reaction centers using the chloroplast translation machinery. Light-induced degradation of D1 relieves the repressive interaction between D1 and the translation activator in the complex, triggering the recruitment of ribosomes to *psbA* mRNA, encoding the D1 protein. This process provides nascent D1 for PSII repair and coordinates D1 biosynthesis with the need for nascent D1 during PSII biogenesis and repair (Chotewutmontri and Barkan 2020).

PSII assembly follows a series of highly ordered steps. First, D2 and cytochrome *b*_*559*_ form a D2–Cyt *b*_*559*_ subcomplex, recruiting another pre-D1 complex consisting of D1 precursor (pD1) and PsbI as well as numerous translation and assembly chaperones to form a transient intermediate subcomplex called the reaction center (RC) complex (Komenda et al. 2004). During the formation of the RC complex, carboxyl terminal peptidase (CtpA) processes the C terminus of the D1 precursor protein to yield mature D1. The RC complex is then converted into an RC47 complex, which is formed by the RC complex and the inner antenna protein CP47 pre-complex containing low molecular mass (LMM) PSII subunits such as PsbH, PsbT, and PsbM. RC47 is bound by Psb28. Subsequently, RC47 sequentially incorporates the inner antenna protein CP43 pre-complex containing the assembly factor Psb27 (also named low PSII accumulation 19 [LPA19]) and LMM subunits, such as PsbK and Psb30, to form a monomeric PSII (Johnson and Pakrasi 2022; Nickelsen and Rengstl 2013). Following the assembly of CP43, the OEC and additional LMM subunits, such as PsbW, bind to monomeric PSII, generating a new monomeric PSII complex. Finally, the repaired PSII monomer migrates back into the stacked membrane, dimerizes with the help of LMM subunits, such as PsbI and PsbM, and combines with LHCII to form a functional PSII–LHCII supercomplex (Johnson and Pakrasi 2022; Nickelsen and Rengstl 2013; Nixon et al. 2010).

Numerous auxiliary nuclear factors are involved in the assembly of the PSII complex (Lu 2016). The plastid-localized PSII assembly factors high chlorophyll fluorescence244 (HCF244), one helix protein1 (OHP1), and OHP2 are thought to form a transient complex that functions in PSII assembly and the translational activation of *psbA* (Chotewutmontri et al. 2020; Hey and Grimm 2018; Li et al. 2019). The chloroplast pentatricopeptide repeat (PPR) protein low photosynthetic efficiency1 (LPE1) interacts with HCF173 and participates in the translational regulation of *psbA* mRNA in a redox-dependent manner (Jin et al. 2018). cyclophilin38 (CYP38) is thought to play a critical role in the correct folding of D1 and the successful assembly of PSII supercomplexes in *Arabidopsis*. *cyp38* mutants are highly susceptible to photoinhibition (Fu et al. 2007; Sirpiö et al. 2008). HCF243 acts as a cofactor to maintain D1 protein stability and to promote the assembly of the PSII complex (Zhang et al. 2011). photosynthesis affected mutant68 (PAM68), an integral thylakoid protein, affects the maturation and stability of newly synthesized D1 and promotes the transition from the RC assembly state to larger PSII assembly complexes (Armbruster et al. 2010). HCF136/YCF48 also mediates the stabilization of D1 and assembly of PSII RC complexes (Chotewutmontri and Barkan 2020; Chotewutmontri et al. 2020; Komenda et al. 2008). A recent study revealed that decreased electron transport at PSII (DEAP2) works in concert with PAM68 to regulate the rapid progression from the RC to RC47 (Keller et al. 2023). LPA2 is required for PSII assembly and proper function (Cecchin et al. 2021). lumen thiol oxidoreductase1 (LTO1), a membrane-embedded disulfide bond–forming catalyst, mediates the assembly of the OEC into PSII (Karamoko et al. 2011). FK-506 binding protein 20-2 (FKBP20-2) functions in the accumulation of the PSII supercomplex, as *fkbp-20* mutants in *Arabidopsis* are more susceptible to photodamage under high-light conditions and accumulate more PSII monomer/dimers than the wild type (Lima et al. 2006). Another protein involved in the formation of the PSII supercomplex, Psb33, is thought to mediate the interaction between the PSII core complexes and LHCII (Fristedt et al. 2015). TROL2 forms an assembly cofactor complex with LPA2 and interacts with small PSII subunits to facilitate PSII complex assembly (Li et al. 2023). Recently, cryo-electron microscopy has been used to analyze PSII structure and revealed the location and binding properties of assembly factors as well as the induced structural changes that protect the not-fully-assembled PSII from photodamage, providing a structural basis for understanding PSII assembly (Huang et al. 2021; Johnson and Pakrasi 2022; Xiao et al. 2021; Zabret et al. 2021).

### Regulation of gene expression

Regulation of gene expression is another important aspect of plant responses and acclimation to high light (Huang et al. 2019; Suzuki et al. 2015) and several photoreceptors have been implicated in sensing high light and initiating transcriptional responses. The blue light photoreceptor CRYPTOCHROME1 (CRY1) is hypothesized as a high light receptor to mediate the perception of high light (Allorent and Petroutsos 2017; Li et al. 2009; Liu et al. 2022; Shaikhali et al. 2012). Transcription factors, such as elongated hypocotyl5 (HY5), ABA insensitive4 (ABI4), MYBs, and basic helix-loop-helix (bHLH), further synergistically regulate the expression of photoprotective genes (Jiang et al. 2020; Li et al. 2009; Shi et al. 2022). In response to high light, plant cells inhibit the transcription of genes encoding antenna proteins while activating the transcription of genes encoding ROS-scavenging enzymes and anthocyanin biosynthetic genes (Huang et al. 2019; Jung et al. 2013; Rossel et al. 2002). The transcriptional response of *Arabidopsis* to high light can be triggered within seconds or minutes of exposure (Suzuki et al. 2015; Vogel et al. 2014). Many photoprotection-related genes are activated at the transcriptional and/or post-transcriptional level to protect the photosynthetic apparatus from high light (Li et al. 2009; Pinnola and Bassi 2018). The abundance of PSII subunits is also increased to promote the rapid biosynthesis and renewal of PSII subunit proteins (Zhang et al. 2021). Alternative nuclear expression of *psbA* can enhance the repair of PSII and improve stress tolerance in plants (Chen et al. 2020). Regulation of genes encoding factors related to the maintenance of PSII function is also critical for maintaining photosynthetic efficiency and protecting PSII under high-light conditions (Li et al. 2020).

Epigenetic regulation also plays an important role in regulating gene expression. High light induces significant changes in the N^6^-methyladenosine (m^6^A) modification of transcripts for chloroplast/photosynthetic genes in plant (Vicente et al. 2023). The m^6^A modification of photoprotection-related transcripts mediated by VIRILIZER (VIR) was shown to regulate the expression of these genes via multiple post-transcriptional steps, such as affecting their stability or translational efficiency (Zhang et al. 2022b). Furthermore, the expression levels of certain genes in plants are altered via DNA methylation. Redox signals arising in chloroplasts regulate DNA methylation levels, which play a major role in the transgenerational embedding of stress tolerance memory in plants (Foyer 2018).

## Conclusion and future perspectives

Plants have developed three main lines of defense that allow them to respond to excess light in a constantly changing environment: limiting exposure, photoprotection, and PSII repair. Limiting exposure includes leaf and chloroplast movement and the filtering of harmful radiation via secondary metabolites. Photoprotection involves ROS scavenging, the timely dissipation of excessive energy mediated by NPQ, CET, and photorespiration. The last line of defense, PSII repair, repairs the damage caused by high light. These photoprotective mechanisms function together to maintain a relatively stable photosynthetic activity under high-light conditions (Fig. 1).

Although we have gained a basic understanding of the strategies plants employ to adapt to high light, more detailed regulatory mechanisms still need to be elucidated. How do plants transduce signals between the nucleus and various organelles after sensing changes in light intensity? How do numerous auxiliary factors in plants work together to precisely regulate the assembly of the photosystem? In addition to transcriptional and post-transcriptional regulation, does post-translational modification of photosynthesis-related proteins participate in the response to high light, and what is its regulatory mechanism? Future research will reveal these answers and deepen our understanding of the mechanisms of plant responses to high light.

The global population is predicted to increase to approximately 10 billion people in the next 30 years. Producing enough food to meet the needs of this population is one of the greatest challenges of this century. With a more extreme global climate, plants are suffering from more and more environmental stresses, which have a major impact on crop production. To avoid reductions in crop yield caused by high light, genetic engineering approaches can be used to optimize the photoprotection capabilities of crops and improve light-use efficiency under light stress conditions. It is also possible to expand the planting area by genetically engineering shade commercial crops or medicinal plants. It will be necessary to weigh the possible imbalance between the beneficial effects of genetic manipulation for improving plant growth in suboptimal environments and the limited carbon gain under optimal conditions, as well as the complexity of plant growth environments. Therefore, more rigorous measurements and analysis under natural conditions are necessary.

## Data Availability

Data sharing not applicable to this article as no datasets were generated or analyzed in the study.
